# Molecular characterization and expression analysis of five different elongation factor 1 alpha genes in the flatfish Senegalese sole (*Solea senegalensis *Kaup): Differential gene expression and thyroid hormones dependence during metamorphosis

**DOI:** 10.1186/1471-2199-9-19

**Published:** 2008-01-30

**Authors:** Carlos Infante, Esther Asensio, José Pedro Cañavate, Manuel Manchado

**Affiliations:** 1 IFAPA Centro *El Toruño*, Junta de Andalucía, Camino Tiro de pichón s/n, 11500 El Puerto de Santa María, Cádiz, Spain

## Abstract

**Background:**

Eukaryotic elongation factor 1 alpha (eEF1A) is one of the four subunits composing eukaryotic translation elongation factor 1. It catalyzes the binding of aminoacyl-tRNA to the A-site of the ribosome in a GTP-dependent manner during protein synthesis, although it also seems to play a role in other non-translational processes. Currently, little information is still available about its expression profile and regulation during flatfish metamorphosis. With regard to this, Senegalese sole (*Solea senegalensis*) is a commercially important flatfish in which *eEF1A *gene remains to be characterized.

**Results:**

The development of large-scale genomics of Senegalese sole has facilitated the identification of five different *eEF1A *genes, referred to as *SseEF1A1*, *SseEF1A2*, *SseEF1A3*, *SseEF1A4*, and *Sse42Sp50*. Main characteristics and sequence identities with other fish and mammalian eEF1As are described. Phylogenetic and tissue expression analyses allowed for the identification of *SseEF1A1 *and *SseEF1A2 *as the Senegalese sole counterparts of mammalian *eEF1A1 *and *eEF1A2*, respectively, and of *Sse42Sp50 *as the ortholog of *Xenopus laevis *and teleost *42Sp50 *gene. The other two elongation factors, *SseEF1A3 *and *SseEF1A4*, represent novel genes that are mainly expressed in gills and skin. The expression profile of the five genes was also studied during larval development, revealing different behaviours. To study the possible regulation of *SseEF1A *gene expressions by thyroid hormones (THs), larvae were exposed to the goitrogen thiourea (TU). TU-treated larvae exhibited lower *SseEF1A4 *mRNA levels than untreated controls at both 11 and 15 days after treatment, whereas transcripts of the other four genes remained relatively unchanged. Moreover, addition of exogenous T4 hormone to TU-treated larvae increased significantly the steady-state levels of *SseEF1A4 *with respect to untreated controls, demonstrating that its expression is up-regulated by THs.

**Conclusion:**

We have identified five different *eEF1A *genes in the Senegalese sole, referred to as *SseEF1A1*, *SseEF1A2*, *SseEF1A3*, *SseEF1A4*, and *Sse42Sp50*. The five genes exhibit different expression patterns in tissues and during larval development. TU and T4 treatments demonstrate that *SseEF1A4 *is up-regulated by THs, suggesting a role in the translational regulation of the factors involved in the dramatic changes that occurs during Senegalese sole metamorphosis.

## Background

G proteins constitute a large superfamily of regulatory proteins that show a high similarity in primary structure and organization of the functional center [1-3]. They all share a common GTPase cycle, being turned on by binding GTP and off by hydrolyzing GTP to GDP. Additional regulatory proteins are often required to induce the conformational changes that occur during this cycle: guanine nucleotide exchange factors, which catalyze release of bound GDP and promote its replacement by GTP, and GTPase-activating proteins, which accelerate GTP hydrolysis. Such transition between active and inactive forms allows for them to serve as molecular switches and to make G proteins suitable for the regulation of a wide range of cellular processes such as signal transduction, cytoskeletal reorganizations, vesicular transport and protein synthesis [4]. The superfamily of G proteins includes three main classes: Ras-like GTPases, G_α _subunits of heterotrimeric G proteins, and the translation elongation factors.

The eukaryotic translation elongation factor 1 alpha, currently termed eEF1A, is a member of the G protein family, and one of the four subunits that compose the eukaryotic elongation factor 1 [5,6]. It is the second-most abundant protein after actin, comprising 1–3% of the total protein content in normal growing cells [7,8]. In its GTP-liganded form, eEF1A catalyzes the first step of the elongation cycle during peptide synthesis, interacting with aminoacyl-tRNA to bring it to the acceptor site of the ribosome [6,9]. Following hydrolysis of the GTP, the complex eEF1A·GDP is released from the ribosome and further converted to the active GTP-bound form by elongation factor 1 beta, which acts as a guanine nucleotide exchange factor for eEF1A. Apart from its key role in protein translation, other functions have been reported as well. eEF1A has been characterized as an actin binding protein with the ability to bundle F actin [10,11] displaying microtubule severing [12] and stabilizing [13] activities, although the biological significance of these interactions is still unclear. In addition, eEF1A is thought to play a role in mediating signal transduction, as it has been shown to interact with PLCγ [14], and to be an activator of phosphoinositol 4-kinase [11,15], an enzyme belonging to the signal transduction cascade activated by growth factors. Furthermore, an altered expression of eEF1A has been found in several kinds of tumors [16-20]. It has been also demonstrated that eEF1A is required for the degradation of N^α^-acetylated proteins probably acting as a ubiquitin C-terminal hydrolase, thus rendering the substrate more easily degraded by the proteasome or by reducing the correct folding of the protein/ubiquitins complex for the subsequent proteolytic degradation [21,22]. Finally, upregulation of eEF1A mediated either by p53 [23,24] or in response to oxidative stress [25,26] seems to be involved in apoptosis. It is likely that this broad diversity of functions may explain why eEF1A is such a well-conserved protein in eukaryotic organisms.

Eukaryotes possess a variable number of *eEF1A *genes. In plants from 10 to 15 *eEF1A *genes have been described in maize [27], and nine in cotton [28]. In fungi, the yeast *Saccharomyces cerevisiae *contains two *eEF1A *genes [29,30], and *Mucor racemosus *has three [31]. In animals, the fruit fly *Drosophila melanogaster *contains two genes, one of which is found to be expressed only in certain stages of development [32]. Three *eEF1A *genes in *Xenopus laevis *are expressed at different developmental stages: the oocyte form (*eEF-1αO*), present in male and female germ cells, the somatic form (*eEF-1αS*), hardly detectable in oocytes but present in embryos and adult cells, and the *42Sp50 *form, detected only in oocytes [33-35]. In the mammalian genome many *eEF1A*-like *loci *exist, most of which appear to be pseudogenes [36-38]. In fact, only two of these sequences are currently known to be actively expressed in human [39,40], mouse [41,42], rat [43-46], and rabbit [47,48]. The two eEF1A protein isoforms encoded by these genes are currently termed eEF1A1 and eEF1A2, and display a quite different expression pattern in tissues. Whereas eEF1A1 is expressed in all tissues, eEF1A2 is only present in brain, heart, and skeletal muscle, which are tissues composed of cells locked in a state of nonproliferation [40,45,49,50]. In teleosts, only one actively transcribed eEF1A gene has been reported in *Danio rerio *[51], *Sparus aurata *[52], *Oreochromis niloticus *[53], and *Oryzias latipes *[54]. In flatfish, partial sequences of one expressed *eEF1A *gene have been described in *Scophthalmus maximus *[55], *Paralichthys olivaceus *[56], and *Paralichthys lethostigma *[57].

Senegalese sole, *Solea senegalensis *(Pleuronectiformes: Soleidae), is a commercially important flatfish. This species undergoes metamorphosis from 12 to 19 days post hatch (DPH) during larval development. The process involves jaw and head restructuring, eye migration from the left to the right side, and a change from a symmetrical to an asymmetrical body shape [58]. These drastic morphological changes have been shown to be regulated by thyroid hormones (THs) in flatfish [59,60]. Nevertheless, few data are available about the biological role of eEF1A in flatfish metamorphosis. In fact, only in *Scophthalmus maximus *this question has been somehow addressed. In this species, coincident T4 and *eEF1A *expression peaks are detected at the climax of metamorphosis [55]. Nevertheless, the gene expression pattern of Senegalese sole *eEF1A *during larval development and in tissues, and its dependence on THs, remains to be elucidated.

Large-scale genomics of Senegalese sole has allowed for the availability of a high number of EST sequences. In this study, we have identified and characterized five different *eEF1A *genes, referred to as *SseEF1A1*, *SseEF1A2*, *SseEF1A3*, *SseEF1A4*, and *Sse42Sp50*. The main sequence features are described hereinafter. A phylogenetic analysis was carried out to identify putative ortholog sequences. Gene expression profiles during larval development and in tissues from juvenile soles were explored using real-time PCR. Additionally, thiourea (TU) and T4 treatments were carried out in order to reveal the possible dependence of all *SseEF1A *expressions on THs.

## Results

### Molecular characterization of Senegalese sole eEF1A genes

Five Senegalese sole *eEF1A *genes, referred to as *SseEF1A1*, *SseEF1A2*, *SseEF1A3*, *SseEF1A4*, and *Sse42Sp50*, were identified after EST analysis of a normalized cDNA library constructed from different larval stages (pre-, meta- and  post-metamorphosis), undifferentiated gonads, and six adult tissues (testis,  ovary, stomach, intestine, liver and brain). A total of 69, 10, 3, 1 and 4 clones were identified as *SseEF1A1*, *SseEF1A2*, *SseEF1A3*, *SseEF1A4 *and *Sse42Sp50*, respectively [DDBJ:AB326302 to AB326306]. Sequencing of specific PCR products amplified from a pre-metamorphic library using Senegalese sole *eEF1A*-specific and the universal T3 and T7 primers confirmed sequences for all *SseEF1A loci*.

Main features of the cDNAs are summarized in Table 1. The cDNA lengths ranged between 1,523 and 1,743 nucleotides (nt) for *SseEF1A3 *and *SseEF1A1*, respectively. All of them contained a short 5'-untranslated region (between 31–73 nt) followed by an open reading frame of 1,365 nt (455 codons; positions 74–1,438 for *Sse42Sp50*), 1,386 nt (462 codons; positions 48–1,433, 35–1,420, and 32–1,417 for *SseEF1A1*, *SseEF1A3*, and *SseEF1A4*, respectively), and 1,389 nt (463 codons; positions 40–1,428 for *SseEF1A2*). The 3'-untranslated region varied between 103 (*SseEF1A3*) and 310 (*SseEF1A1*) nt long and included one (*SseEF1A2*, *SseEF1A4 *and *Sse42Sp50*) or two (*SseEF1A1 *and *SseEF1A3*) canonical polyadenylation signals (AATAAA), and a short oligo-A tail. The nucleotide sequences surrounding the ATG initiator codon fit well to the consensus sequence of Kozak [61], including A (*SseEF1A1*, *SseEF1A2*, *SseEF1A3 *and *SseEF1A4*) or G (*Sse42Sp50*) at position -3 and G at position +4 (considering position +1 the A from the ATG start codon). The predicted molecular weights and  isoelectric points of the translated amino acid sequences of Senegalese sole  eEF1A genes ranged between 49.86-50.53 kDa and 9.13-9.69, respectively.

**Table 1:** Main features of the cDNAs encoding for SseEF1As.

Locus	cDNA length (nt)	5'-UTR length (nt)	Coding sequence	3'-UTR length (nt)	3' polyadenylation signal
*SseEF1A1*	1,743	47	48–1,433	310	1,724–1,729 1,730–1,735
*SseEF1A2*	1,668	39	40–1,428	239	1,650–1,655
*SseEF1A3*	1,523	34	35–1,420	103	1,495–1,500 1,504–1,509
*SseEF1A4*	1,603	31	32–1,417	186	1,582–1,587
*Sse42Sp50*	1,665	73	74–1,438	227	1,645–1,650

**Figure 1:**
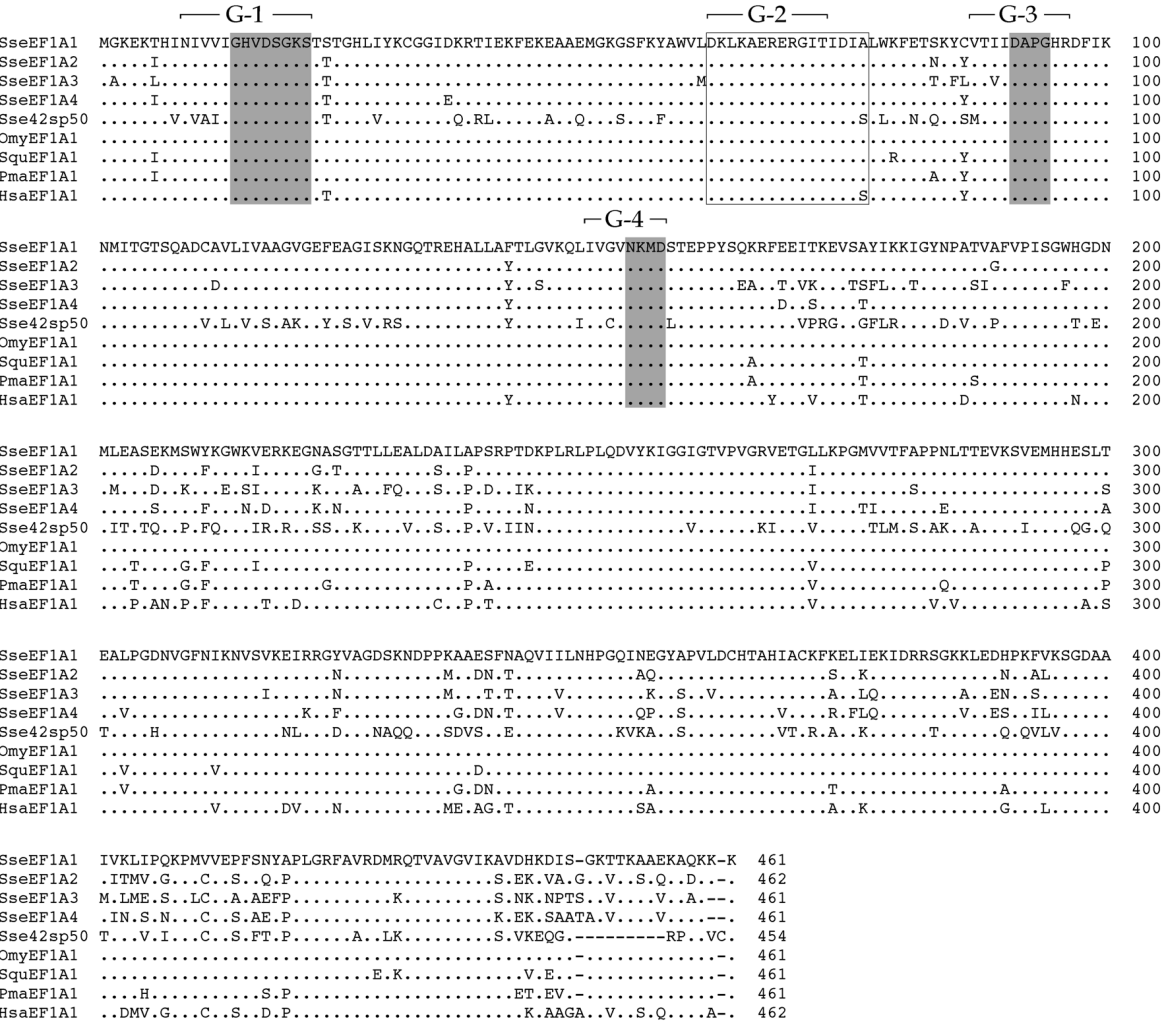
Comparison of the primary structure of SseEF1As and various others eEF1A proteins (see Table 3). The alignment was performed using MegAlign software. Dots represent identity with SseEF1A1, and dashes represent gaps. G1 to G4 indicate the critical regions involved in GDP/GTP exchange and GTP hydrolysis. The consensus sequence composed of the three consensus elements GXXXXGK (G^14^-K^20^), DXXG (D^91^-G^94^), and NKXD (N^153^-D^156^) present in the GTP-binding domain is shaded in grey. The GTP-binding elongation factor signature corresponding to amino acids 61 to 76 is boxed.

All SseEF1As possessed the characteristic regions G1 to G4 critical in GDP/GTP exchange, GTP-induced conformational change and GTP hydrolysis (Figure 1) [1]. The three consensus elements GXXXXGK (G^14^-K^20^), DXXG (D^91^-G^94^) and NKXD (N^153^-D^156^) [2] were identified in the GTP-binding domain of the Senegalese sole polypeptides. In addition, the predicted amino acid sequences were scanned against the PROSITE database of sequence motifs. The conserved motif DKLKAERERGITIDI(A/S) was identified at positions 61 to 76 in each of SseEF1As as the GTP-binding elongation factor signature (Figure 1).

*SseEF1A *genes shared high sequence homology at nucleotide level in the coding region (62.9–88.6% identity) and at amino acid level (66.7–89.8% identity). A multiple alignment was performed with the DNA (Table 2; below diagonal) and amino acid sequences (Table 2; above diagonal) of *eEF1A *from other vertebrates (see Table 3). As observed for Senegalese sole sequences, the identities obtained in all the pairwise comparisons were lower at DNA than at amino acid level, indicating that the highest sequence variation was at the third codon positions. Identities of SseEF1A1 and SseEF1A2 amino acid sequences were higher than those determined for SseEF1A3 and SseEF1A4 with regard to eEF1A from the other species. By the other side, 42Sp50 sequences exhibited high identities, with values above 87% in the comparisons performed.

**Table 2:** Percentage of amino acid (above diagonal) and nucleotide (below diagonal) sequence identity among Senegalese sole elongation factors and several other eEF1As (see Table 3) as calculated using MegAlign.

	SseEF1A1	SseEF1A2	SseEF1A3	SseEF1A4	Sse42sp50	OlaEF1A1	Ola42sp50	TruEF1A1	Tru42sp50	HsaEF1A1	HsaEF1A2
	
SseEF1A1	-	89.8	82.0	86.1	69.8	95.4	67.5	90.0	67.5	87.4	84.8
SseEF1A2	88.6	-	84.4	89.2	71.6	90.2	69.9	92.0	70.1	90.0	89.0
SseEF1A3	81.6	83.5	-	82.6	66.7	82.4	65.9	81.7	66.2	80.7	81.1
SseEF1A4	85.9	88.5	83.0	-	68.7	86.8	67.5	87.2	67.7	85.5	84.4
Sse42sp50	65.5	65.4	62.9	64.2	-	69.8	91.6	69.6	91.0	71.1	70.0
OlaEF1A1	90.0	86.0	79.7	82.0	64.2	-	67.7	91.3	67.5	88.1	84.6
Ola42sp50	62.7	63.6	60.2	62.5	82.9	63.0	-	67.4	87.7	69.0	68.2
TruEF1A1	84.6	84.8	78.4	81.8	64.6	84.8	62.2	-	68.3	88.3	87.4
Tru42sp50	64.1	65.5	61.8	63.9	81.6	64.4	78.7	63.6	-	69.5	69.3
HsaEF1A1	77.0	77.7	71.9	75.2	63.8	77.0	62.6	76.7	60.9	-	92.4
HsaEF1A2	76.9	80.0	71.6	75.8	64.9	76.7	63.9	77.5	67.3	76.4	-

**Table 3:** Vertebrate eEF1A sequences used in this study. The abbreviated name, description, accession number and species are indicated in each case.

Name	Description	Accession number	Species
ArtEF1A	eEF1A	X03349	*Artemia *sp.
DreEF1A	eEF1A	X77689	*Danio rerio*
Dre1	eEF1A family	ENSDARG00000006838	
Dre2	eEF1A family	ENSDARG00000020850	
Dre3	eEF1A family	ENSDARG00000039502	
Dre4	eEF1A family	ENSDARG00000053313	
Dre5	eEF1A family	ENSDARG00000053319	
Dre6	eEF1A family	ENSDARG00000052832	
Dre7	eEF1A family	ENSDARG00000059223	
DmeEF1A	eEF1A	NM_079872	*Drosophila melanogaster*
GgaEF1A1	eEF1A1	NM_204157	*Gallus gallus*
GgaEF1A2	eEF1A2	XM_001233517	
Gac1	eEF1A family	ENSGACG00000002143	*Gasterosteus aculeatus*
Gac2	eEF1A family	ENSGACG00000002175	
Gac3	eEF1A family	ENSGACG00000002182	
Gac4	eEF1A family	ENSGACG00000004505	
Gac5	eEF1A family	ENSGACG00000018090	
HhiEF1A	eEF1A	EB174049	*Hippoglossus hippoglossus*
HsaEF1A1	eEF1A1	NM_001402	*Homo sapiens*
HsaEF1A2	eEF1A2	NM_001958	
Hsa1	eEF1A family	ENSG00000183920	
Hsa2	eEF1A family	ENSG00000185637	
MmuEF1A1	eEF1A1	NM_010106	*Mus musculus*
MmuEF1A2	eEF1A2	NM_007906	
Mmu1	eEF1A family	ENSMUSG00000052588	
Mmu2	eEF1A family	ENSMUSG00000068225	
OmyEF1A	eEF1A	AF498320	*Oncorhynchus mykiss*
OniEF1A	eEF1A	AB075952	*Oreochromis niloticus*
OcuEF1A1	eEF1A1	NM_001082339	*Oryctolagus cuniculus*
OcuEF1A2	eEF1A2	NM_001082031	
Ocu1	eEF1A family	ENSOCUG00000013034	
OlaEF1A	eEF1A	AB013606	*Oryzias latipes*
Ola42Sp50	42Sp50	AF128815	
Ola1	eEF1A family	ENSORLG00000007585	
Ola2	eEF1A family	ENSORLG00000002258	
Ola3	eEF1A family	ENSORLG00000019066	
PmaEF1A	eEF1A	AY190693	*Pagrus major*
PflEF1A	eEF1A	EC379379	*Platichthys flesus*
RnoEF1A1	eEF1A1	NM_175838	*Rattus norvegicus*
RnoEF1A2	eEF1A2	NM_012660	
Rno1	eEF1A family	ENSRNOG00000004012	
Rno2	eEF1A family	ENSRNOG00000012863	
Rno3	eEF1A family	ENSRNOG00000015106	
Rno4	eEF1A family	ENSRNOG00000024179	
Rno5	eEF1A family	ENSRNOG00000027199	
Rno6	eEF1A family	ENSRNOG00000027390	
SsaEF1A	eEF1A	AF321836	*Salmo salar*
SmaEF1A		AF467776	*Scophthalmus maximus*
SquEF1A	eEF1A	AB032900	*Seriola quinqueradiata*
SauEF1A	eEF1A	AF184170	*Sparus aurata*
TruEF1A		SINFRUG00000160135	*Takifugu rubripes*
Tru42Sp50	42Sp50	SINFRUG00000134533	
Tru1	eEF1A family	SINFRUG00000124608	
Tni42Sp50	42Sp50	GSTENG00031816001	*Tetraodon nigroviridis*
Tni1	eEF1A family	GSTENG00003643001	
Tni2	eEF1A family	GSTENG00018671001	
Tni3	eEF1A family	GSTENG00031816001	
XlaEF1A	eEF1A	X55324	*Xenopus laevis*
Xla42Sp50	42Sp50	Z19545	

**Figure 2:**
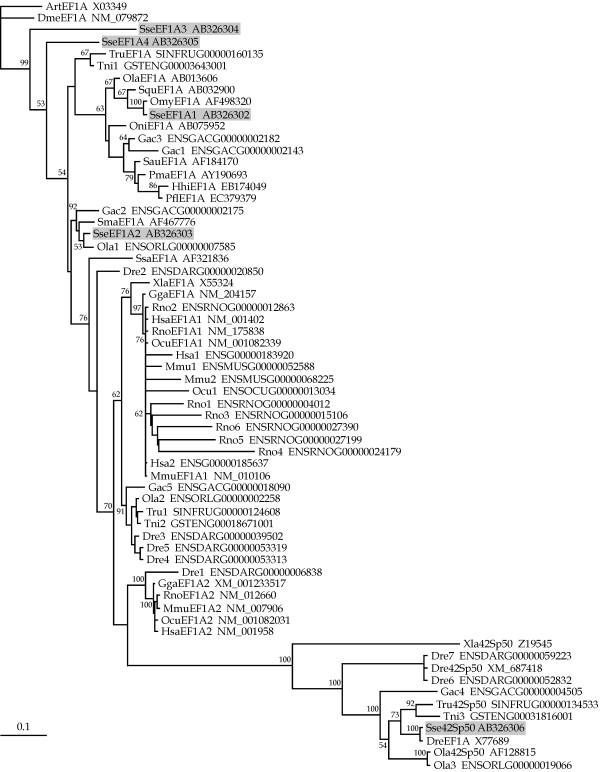
Phylogenetic relationships among SseEF1As and a wide range of vertebrate eEF1As (see Table 3) using the maximum likelihood method. *D. melanogaster* and *Artemia* sp elongation factor 1  alpha proteins were used as outgroups to root tree. Only bootstrap values higher than 50% are indicated on each branch. The scale for branch length (0.1 substitutions/site) is shown below the tree.

**Figure 3:**
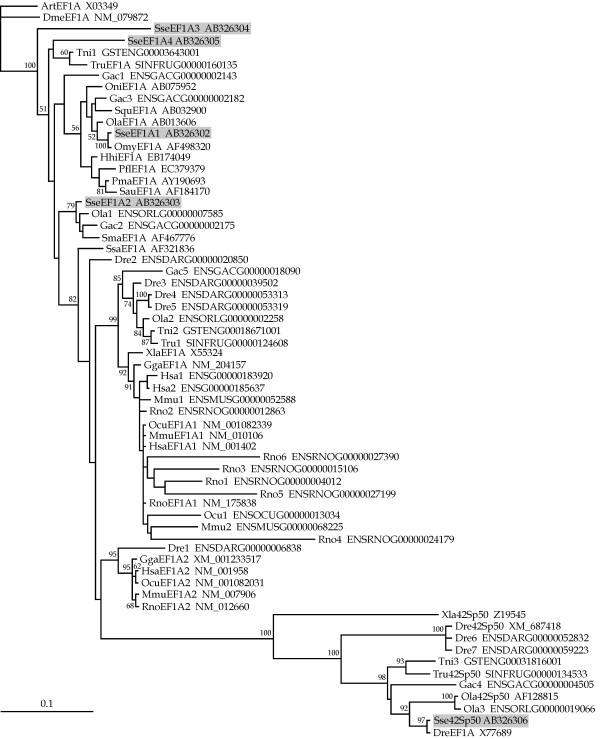
Phylogenetic relationships among SseEF1As and a wide range of vertebrate eEF1As (see Table 3) using the neighbor-joining method. *D. melanogaster* and *Artemia* sp elongation factor 1  alpha proteins were used as outgroups to root tree. Only bootstrap values higher than 50% are indicated on each branch. The scale for branch length (0.1 substitutions/site) is shown below the tree.

The phylogenetic relationships of the five SseEF1As as well as the predicted protein sequence of other vertebrate *eEF1A *genes were determined using the maximum likelihood (ML) and neighbor-joining (NJ) methods. The ProtTest analysis [62] determined the JTT + G model as being the bestfit model of amino acid sequence evolution (-ln*L *= 8,642.63) with a gamma shape value (four rate categories) of 0.69. These settings were also employed for the NJ analysis. The topology of the trees obtained with these parameters are depicted in Figure 2 (ML) and Figure 3 (NJ), with *Drosophila melanogaster *[GeneBank:NM_079872) and *Artemia *[EMBL:X03349] eEF1A proteins used as outgroups. Complex evolutionary relatedness was obtained. SseEF1A1 was linked together with other teleost eEF1As in a consistent clade (63 and 56% of bootstrap value for ML and NJ, respectively). SseEF1A2 was clustered in a highly supported branch (92 and 79% of bootstrap value for ML and NJ, respectively) with turbot eEF1A as well as with two other teleost sequences from *Oryzias latipes *and *Gasterosteus aculeatus*. In addition, all 42Sp50 proteins were grouped together in a highly consistent ramification (100% bootstrap support in ML and NJ trees). Interestingly, both SseEF1A3 and SseEF1A4 appeared in a more basal position of both trees, and no consistent clustering with any of the sequences added was observed.

### Expression levels of SseEF1A genes in tissues

**Figure 4:**
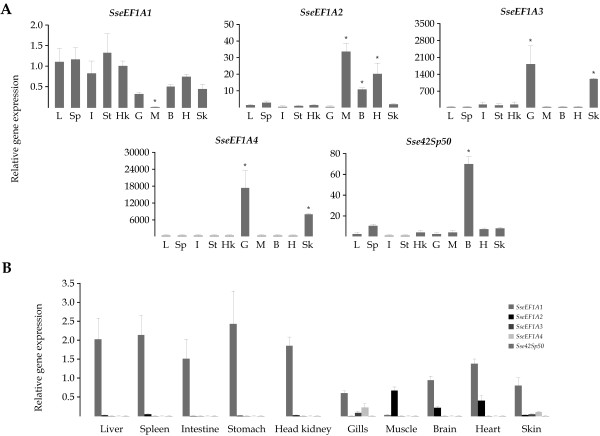
A) Relative expression levels in tissues of the five *SseEF1A *genes. Expression values were normalized to those of *Ubiquitin*. Data were expressed as the mean fold change (mean ± SEM, n = 3) from the calibrator group (liver). Values marked with an asterisk are significantly different from liver values (*P *< 0.05). L: liver, Sp: spleen, I: intestine, St: stomach, Hk: head-kidney, G: gills, M: skeletal muscle, B: brain, H: heart, Sk: skin. B) Comparison of the relative levels of *SseEF1A *transcripts in tissues. Data are expressed as the ratio (calculated using 2^-(ΔCt)^) of target mRNA to *Ubiquitin *mRNA.

Steady-state levels of the five *SseEF1A *transcripts were quantitated in liver, spleen, intestine, stomach, head-kidney, gills, skeletal muscle, brain, heart, and skin from juvenile soles (Figure 4A). Relative gene expression levels were normalized by measuring *Ubiquitin *gene and expressed relative to liver. All *SseEF1A *genes were found in detectable amounts in the tissues examined. *SseEF1A1 *transcripts were quite similar in all tissues examined except in muscle (60-fold lower than in liver; *P *< 0.05). *SseEF1A2 *reached the highest expression levels in skeletal muscle, heart, and brain (28, 17, and 9-fold higher than liver, respectively; *P *< 0.05). *SseEF1A3 *and *SseEF1A4 *showed very similar expression patterns, as they were strongly expressed in gills (750 and 13,000-fold higher than liver, respectively; *P *< 0.001) and skin (500 and 6,000-fold higher than liver, respectively; *P *< 0.001). Finally, *Sse42Sp50 *was expressed at a relatively high level in brain (32-fold higher than liver; *P *< 0.001).

Although they exhibited differential expression profiles, we calculated the relative amount of the five *SseEF1A *mRNA levels in the 10 tissues examined (Figure 4B). As a whole, *SseEF1A1 *transcripts were the most abundant with 60, 2,000, 62,000, and 35,000-fold higher overall mean expression ratios than *SseEF1A2*, *SseEF1A3*, *SseEF1A4*, and *Sse42Sp50*, respectively. Nevertheless, *SseEF1A2 *showed the highest values in muscle (20-fold higher than *SseEF1A1*), and it was only 4 and 3-fold lower expressed than *SseEF1A1 *in brain and heart, respectively. *SseEF1A3 *and *SseEF1A4 *reached relatively important expression levels in gills (7 and 3-fold lower than *SseEF1A1*, respectively) and skin (14 and 8-fold lower than *SseEF1A1*, respectively). Finally, *Sse42Sp50 *was expressed at very low amounts in all tissues.

### Expression levels and regulation during larval development

**Figure 5:**
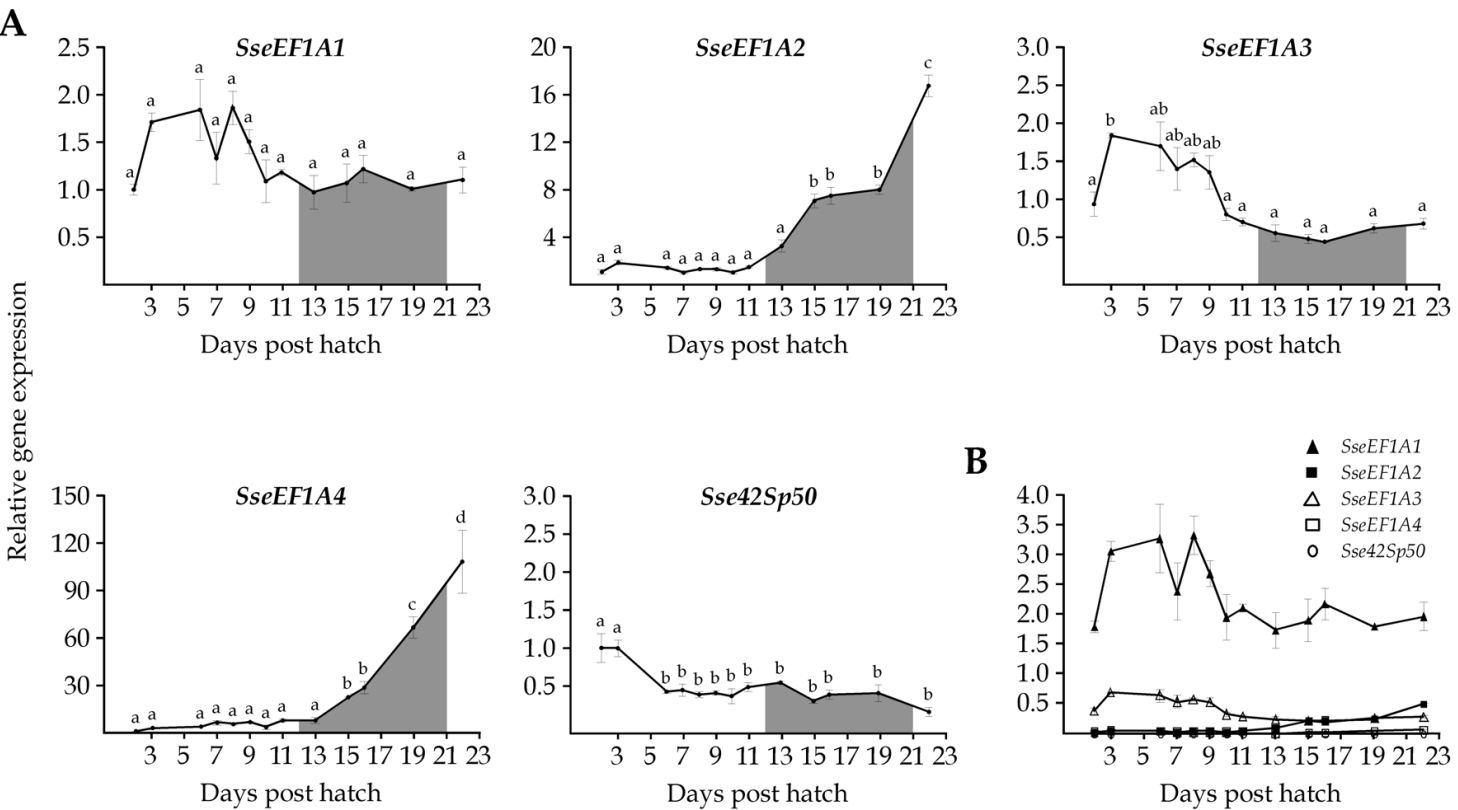
A) Relative *SseEF1A *expression levels during larval development (from 2 to 22 DPH) in Senegalese sole. Expression values were normalized to those of *GAPDH2*. Data are expressed as the mean fold change (mean ± SEM, n = 3) from the calibrator group (2 DPH). Different letters denote days that are significantly different (*P *< 0.05) analyzed by ANOVA followed by a Tukey test. The interval for the metamorphic process is shaded. B) Comparison of the relative levels of *SseEF1A *transcripts during larval development. Data are expressed as the ratio (calculated using 2^-(ΔCt)^) of target mRNA to *GAPDH2 *mRNA.

Expression patterns of *SseEF1A *genes during larval development (from 2 to 22 DPH) were also determined. Data were normalized to the housekeeping gene glyceraldehyde-3-phosphate dehydrogenase (*GAPDH2*) [63], and further expressed relative to 2 DPH. All transcripts were detected very early at 2 DPH (Figure 5A). Nevertheless, they displayed different expression profiles during development. *SseEF1A1 *transcripts remained relatively constant with no significant changes between pre-metamorphosis and metamorphosis. A similar expression pattern was observed for *SseEF1A3*, although a significant peak in mRNA levels was observed at first feeding (1.89-fold; *P *< 0.05). The expression profile of *SseEF1A2 *and *SseEF1A4 *was quite similar. Both transcripts were constant until 15 DPH, when they increased significantly (7 and 22-fold, respectively; *P *< 0.001), and thereafter they rose progressively until the end of metamorphosis. In relation to *Sse42Sp50*, the highest expression levels were detected at first developmental stages with no significant changes from 6 to 22 DPH. As observed in tissues, *SseEF1A1 *was the most abundantly expressed of all genes during larval development (Figure 5B).

**Figure 6:**
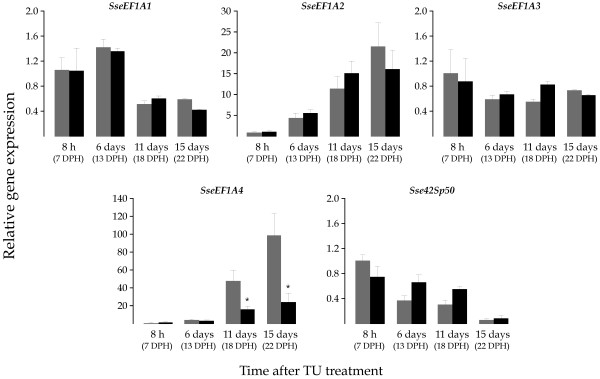
Relative *SseEF1A *expression levels as determined by real-time quantitative PCR in the untreated (grey) and TU-treated (black) groups. Untreated and TU-treated samples were collected for RNA isolation at four different times: 8 hours, and 6 days, 11 days, and 15 days after treatment starting on 7 DPH. Expression values were normalized to those of *GAPDH2*. Data are expressed as the mean fold change (mean ± SEM, n = 3) from the calibrator group (control 8 hours). Values marked with an asterisk are significantly different (*P *< 0.05 or less) from the corresponding untreated control group values.

To study the involvement of THs on the expression of *SseEF1A *genes, 7 DPH larvae were exposed to the goitrogen TU. As a consequence of the TU treatment, the metamorphic process was blocked at S1-S3 stages as determined by the degree of eye migration. No differences in survivability were observed with respect to the untreated control (not shown). mRNA levels for *SseEF1A *genes were quantified in whole larvae pools collected at 8 hours, and 6 days, 11 days, and 15 days after treatment (dat). Untreated control larvae exhibited expression profiles similar to those described above in all cases (Figure 6). No significant differences in gene expression were observed for *SseEF1A1*, *SseEF1A2*, *SseEF1A3 *and *Sse42sp50 *between untreated control and TU-treated larvae. However, TU-treated larvae showed 3 and 4-fold lower (*P *< 0.05) *SseEF1A4 *mRNA levels than untreated controls at both 11 and 15 dat, respectively.

**Figure 7:**
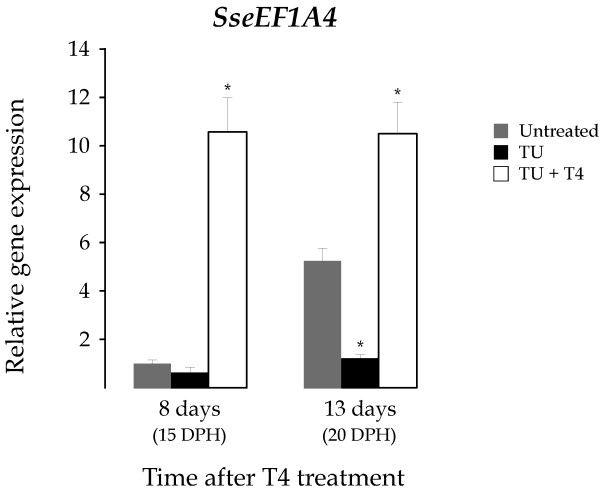
Relative *SseEF1A4 *expression levels as determined by real-time quantitative PCR in the untreated control (grey), and in TU (black) and TU+T4 (white) treated groups. Larvae samples were collected for rRNA isolation at 8 and 13 days after T4 treatment starting 7 DPH. Expression values were normalized to those of *GAPDH2*. Data are expressed as the mean fold change (mean ± SEM, n = 3) from the calibrator group (untreated control day 8). Values marked with an asterisk are significantly different (*P *< 0.05 or less) from the corresponding untreated control group values.

At the sight of these results, we carried out a rescue assay to determine the ability of T4 to revert the TU effect on the expression of *SseEF1A4*. For this purpose, 4 DPH larvae were exposed to TU to ensure the lack of endogenously synthesized T4 at day 7, when the exogenous T4 (100 ppb) was supplied. mRNA levels of *SseEF1A4 *gene were determined in larvae sampled at 8 and 13 days after T4 treatment (datt). The metamorphosis blocking induced by TU was not observed in TU+T4 treated larvae. The expression patterns of untreated and TU-treated larvae were similar to those described above (Figures 5 and 6). Interestingly, TU+T4 treated larvae exhibited significantly higher *SseEF1A4 *transcript levels than untreated (10.5 and 2-fold; *P *< 0.05) and TU-treated (16.8 and 8.7-fold; *P *< 0.05) larvae both at 8 and 13 datt, respectively (Figure 7).

## Discussion

Three rounds of large-scale gene duplications, referred to as 1R, 2R, and 3R or fish-specific genome duplication, have been proposed to take place in the fish evolutionary lineage [64,65]. As a result, several gene copies have been described in teleosts for some group of genes including glycolytic enzymes [63,64], Hox genes [66,67], hormones and their receptors [68,69], and keratins [70]. The majority of gene duplicates have been lost or silenced during evolution but in some instances paralogue genes have picked up new functions (neofunctionalization), or divided the ancestral function between paralogs (subfunctionalization) [71,72]. Such a selective advantage would have avoided loss of functionally active duplicates. In this study, we have obtained the complete cDNA sequence of five distinct Senegalese sole *eEF1A *genes, referred to as *SseEF1A1, SseEF1A2, SseEF1A3, SseEF1A4*, and *Sse42Sp50*. All of them share high identity at both  nucleotide (62.9-88.6%) and amino acid (66.7-89.8%) levels. Amino acid sequence analysis of the predicted polypeptides revealed the presence of the consensus amino acid sequence that identifies a GTP-binding protein [1,2] in all of them with spacing between the characteristic sequence elements GXXXXGK (G^14^-K^20^), DXXG (D^91^-G^94^) and NKXD (N^153^-D^156^) that fell into the expected size (40–80 residues). Furthermore, the five Senegalese sole proteins possessed the characteristic motif that provides a signature for GTP-binding elongation factor. Altogether, these results indicate that *SseEF1A *genes actually encode for functional eEF1A proteins.

Phylogenetic analyses were carried out using a wide range of vertebrate eEF1A predicted proteins. A number of sequences found in the complete genome of several teleosts and mammals belonging to eEF1A family were also included, although it is not known if such sequences are actively transcribed or represent pseudogenes. Phylogeny gives strong support to the classification of *SseEF1A1 *as the Senegalese sole ortholog of teleost *eEF1A*. Furthermore, we can consider that teleost eEF1A, including SseEF1A1, is the fish counterpart of tetrapod eEF1A1. Such a conclusion is based on two main observations. Firstly, the high sequence identities of teleost with tetrapod eEF1A proteins as obtained with the sequences employed in this study, ranging between 86.6–89.9% (not shown). And secondly, gene expression analyses indicate that teleost *eEF1A *[52-54] as well as *SseEF1A *is a highly abundant mRNA transcript that exhibits a relatively uniform distribution across many tissues, as observed in mammalian *eEF1A1 *[40,45,48,49,73]. In contrast, strikingly enough, turbot SmaEF1A as well as the predicted polypeptides of one *O. latipes *and *G. aculeatus *sequences appeared closely linked to SseEF1A2 in a different clade to teleost eEF1A, being even more closely related to mammalian (and avian) eEF1A isoforms. According to the expression pattern in tissues of *SseEF1A2*, we suggest that this fish paralog represents the counterpart to mammalian eEF1A2. In this sense, northern blot and immunoblotting analyses have revealed that expression of mammalian *eEF1A2 *is limited to the terminally differentiated cells of the skeletal muscle, brain, and heart [40,45,48,49,73], the same tissues where *SseEF1A2 *reached the highest expression levels. If this gene is only expressed in the species analyzed here or in a wide range of flatfish or teleosts is a problem that needs to be resolved in future studies. In any case, the presence of these two genes in fish and mammals suggests that such gene duplication was an early evolutionary event. An interesting issue is that, in contrast to teleost, a close phylogenetic relatedness between mammalian eEF1A1 and eEF1A2 isoforms was observed. One possible explanation for this important difference is the increased evolutionary rate that is known to occur in fish genes [74] making sequence divergence between mammalian and fish counterparts comparable to a pair of fish paralogous genes.

The biological roles of mammalian eEF1A isoforms have been widely studied [6,8,75]. At present, it is unclear why there are two isoforms of eEF1A. *In vitro*, the translational elongation activity is similar but there is a difference in the off-rate for GDP as eEF1A2 binds GDP more strongly than GTP, whereas the opposite is true for eEF1A1 [48]. Given that eEF1A2 is expressed specifically in cells that have permanently withdrawn from the cell cycle such as neurons, cardiomyocites and myocites [40,41,48], it has been suggested that eEF1A2 may be involved in pathways of protein synthesis specifically for nonproliferating cells or in the non-translational roles suggested for eEF1A. It seems that eEF1A2 is anti-apoptotic [76], which is closely related with its possible role in tumourigenesis [17,20,77]. If we consider the high degree of structural and functional relatedness existing between eEF1A from different organisms, it is highly probable that fish orthologous genes carry out the same functions as mammalian isoforms.

All 42Sp50 proteins grouped together in a highly consistent cluster both in ML and NJ trees. Although orthologous genes have been described in other teleosts, we could no identify a mammalian counterpart in any of the complete genomes available in databases. Thus, it seems that this gene was lost early in the evolution of tetrapods. Little information is found concerning its possible function. In *X. laevis*, 42Sp50 transcripts have only been detected in oocytes, and the protein interacts with other components of the 42S RNP particle, which serves as storage of 5S rRNA and various tRNAs [34]. Senegalese sole *Sse42Sp50 *was expressed in all tissues examined, but at a highly significant lesser amount than *SseEF1A1*. Phylogeny of SseEF1A3 and SseEF1A4 is more difficult to interpret because it was poorly resolved, mainly due to the fact that no teleost (and no mammalian) sequence significantly clustered with them. Furthermore, we have failed to identify possible counterparts in the complete genome of other teleosts and mammals available in databases. Nevertheless, we argue that both *SseEF1A3 *and *SseEF1A4 *are probably the result of recent gene duplications. Both genes expressed mainly in gills and skin, two tissues involved in respiratory and osmoregulatory processes [78]. It is tempting to assume that they could be involved in the translational regulation of factors playing a role in such important biological activities in the Senegalese sole. And in the case of *SseEF1A4*, an additional role in metamorphosis will be discussed below.

Metamorphosis in flatfish is characterized by the change from a symmetrical, pelagic larva to an asymmetric, benthic juvenile, and by the eye migration from one side of the head to the other. THs have shown to play a key role in flatfish metamorphosis. Important physiological and molecular switches in erythropoiesis, myosin light chains, troponin T, and gill mitochondria-rich cells as well as differentiation of gastric glands and epidermal adult cells, and re-organization of the neural retina skin have been reported to occur during this dramatic process in a THs dependent manner [79-85]. More recently, it has also been reported that THs down-regulate *GAPDH1 *transcripts after metamorphosis climax [63] demonstrating the dependence of the glycolytic pathway on THs. With regard to *eEF1A *an expression peak at the climax of metamorphosis, coincident with a T4 peak, has been detected in turbot [55]. Interestingly, regulation by THs of the germ cell specific EF-1αO gene has been demonstrated during metamorphosis in *X. laevis *[86]. The start of metamorphosis has been associated to a surge of THs that increase their levels until the metamorphic climax, and reduce towards post-climax [59,60]. In this study, the possible regulation of *SseEF1A *genes by THs was analyzed using TU and combined TU+T4 treatments. TU is a blocking agent of THs synthesis that can reduce T4 levels by about 95% [82]. Exogenous treatments with TU as well as other types of thyroid inhibitors have proved useful to study the involvement of THs on metamorphosis in flatfish [59,82,84] and the transcriptional regulation of genes involved in the pituitary-thyroid axis [87,88]. TU-treated larvae exhibited significantly lower *SseEF1A4 *transcripts levels than untreated larvae at late metamorphosis, whereas mRNA levels of the other four genes remained relatively unchanged. In contrast, addition of T4 increased the amount of *SseEF1A4 *transcripts to significantly higher levels than those detected in untreated and TU-treated larvae. These results demonstrate that THs up-regulate either directly or indirectly *SseEF1A4 *expression, and constitute the first report of the dependence on THs of an elongation factor gene during flatfish metamorphosis. Although further research will be required to elucidate the precise role of *SseEF1A4 *in the drastic changes that occur during metamorphosis, it can be hypothesized that *SseEF1A4 *is involved in the translational regulation of the factors that promote an appropriate transition from larval to juvenile stage in response to THs. In fact, elongation factors are directly involved in the regulation of peptide synthesis [89,90]. Yet, other non-translational functions during metamorphosis cannot be ruled out.

## Conclusion

In conclusion, in this work we describe the sequence and main features of five *eEF1A *genes in the Senegalese sole, referred to as *SseEF1A1*, *SseEF1A2*, *SseEF1A3*, *SseEF1A4*, and *Sse42Sp50*. Combined phylogenetic and tissue expression analyses revealed *SseEF1A1 *and *SseEF1A2 *as the putative counterparts of mammalian *eEF1A1 *and *eEF1A2*, respectively, and *Sse42Sp50 *as the ortholog of *X. laevis *and teleost *42Sp50*. In contrast, *SseEF1A3 *and *SseEF1A4 *represent novel genes, as orthologous genes have not been identified neither in fish nor mammals. The five genes exhibited different expression patterns during larval development. TU and T4 treatments demonstrated that *SseEF1A4 *was up-regulated by THs, suggesting a role in the translational regulation of the factors involved in the dramatic changes that occur during Senegalese sole metamorphosis. The regulation and interaction of Senegalese sole elongation factors with other genes are questions that need to be clarified in the future and will help to improve the aquaculture conditions for this species. Wih regard to this, present work constitutes undoubtly a useful framework for further studies.

## Methods

### Source of fish and experimental rearing conditions

All experimental sole larvae were obtained from fertilized eggs collected from breeding tanks, where breeders spawned naturally under environmental conditions. Eggs were incubated at a density of 2,000 eggs L^-1 ^in 300 L cylinder conical tanks with gentle aeration, and water exchange every two hours. Temperature and salinity during all experiments were 20°C and 38 psu, respectively. Newly hatched larvae were transferred to a 400 L tank at an initial density of 45 to 50 larvae L^-1 ^with a 16L:8D photoperiod, and a light intensity of 600–800 lux. Larvae were fed rotifers (*Brachionus plicatilis*) 3 DPH until 9 DPH. Enriched *Artemia *metanauplii were fed to them from 7 DPH until the end of the experiment. Pools of larvae from 2 to 22 DPH (n = 3) were collected, washed with DEPC water, frozen in liquid nitrogen, and stored at -80°C until analysis. Metamorphic stages (S0-S4) were classified according to [58].

Chronic exposure to 30 ppm TU was achieved in 200 L round tanks. TU was added on day 7, and water was kept stagnant for 24 hours. After this exposure, 20% of the water was exchanged daily, with the subsequent addition of the eliminated TU. A second tank with the same characteristics was used to rear untreated control sole larvae. Larvae were initially stocked at a density of 100 individual L^-1^, and lights were kept off until the onset of external feeding on 3 DPH. Fluorescent lamps supplied an illumination of 800 lux on the water surface, while a 16L:8D light cycle was used. Larvae were fed rotifers (*Brachionus plicatilis*) and 48 hours *Artemia *metanauplii, according to the experimental design. The Haptophyceae *Isochrysis galbana *(T-ISO strain) was employed for the enrichment of both live preys, and was also added (2 mg dry weight L^-1 ^d^-1^) to larval tanks during rotifer feed. Daily larval samples were removed for metamorphosis control. Pools of larvae (n = 3) were also collected, washed with DEPC water, frozen in liquid nitrogen, and stored at -80°C until analysis.

For the rescue experiments, culture conditions were the same as described above, but 30 ppm TU was added on day 4 to ensure lack of endogenously synthesized T4 at the commencement of exogenous T4 treatment. At day 7, untreated control larvae were transferred to a pair of 16 L cylinder conical tubes at an initial density of 45 larvae L^-1^. TU-treated larvae were transferred to 4 tubes, two of which were supplemented with 100 ppb T4. After this new exposure, 20% of the water was exchanged every two days, with the subsequent addition of the eliminated TU and TU+T4. Pools of larvae (n = 3) were collected 8 and 13 days after the commencement of T4 treatment, washed with DEPC water, frozen in liquid nitrogen, and stored at -80°C until analysis.

Juvenile Senegalese sole individuals (average weight = 169.60 ± 24.3 g; n = 3) were obtained from IFAPA Centro *El Toruño *facilities (El Puerto Santa María, Cádiz, Spain). They were sacrificed by immersion in tricaine methanesulfonate (MS-222) according to the guidelines on the care and use of fish in research, teaching and testing from the Canadian Council on Animal Care (2005). Head-kidney, liver, testis, brain, heart, skin, skeletal muscle, spleen, intestine, stomach, and gills were rapidly dissected, frozen in liquid nitrogen, and stored at -80°C until use.

### Identification and sequence analyses of elongation factor 1 alpha cDNAs in Senegalese sole

Ten cDNA libraries were constructed from different larval stages and adult tissues of Senegalese sole using the ZAP Express^® ^cDNA Syntesis kit and Zap Express cDNA Gigapack^® ^III Gold Cloning kit (Stratagene) following the manufacturer's protocol (Cerdà et al., in preparation). Libraries were pooled and normalized, and approximately 11,000 randomly selected clones were sequenced from the 3'-end. Expressed sequence tags (ESTs) encoding *eEF1A *genes were identified after EST annotation. Sequences have been deposited with accession numbers [DDBJ:AB326302 to AB326306].

Alignments of the predicted polypeptide sequences were carried out, and the sequence identities calculated by the MegAlign program from the LASERGENE software suite. For this purpose as well as for the ML and NJ phylogenetic analyses, predicted polypeptides of a range of vertebrate *eEF1A *sequences retrieved from GenBank/EMBL/DDBJ were used (Table 3). Additional sequences belonging to the *eEF1A *family were retrieved from ENSEMBL (Table 3). *Drosophila melanogaster *[GeneBank:NM_079872) and *Artemia *[EMBL:X03349] elongation factor 1 alpha sequences were used as outgroups to root ML and NJ trees. The bestfit model of sequence evolution was determined by using the ProtTest *v*1.3 [62]. Phylogenetic analysis was carried out using the PHYLIP package [91] as follows. A bootstrap analysis was performed using SEQBOOT (200 replicates for ML; 1000 replicates for NJ). Data were then analyzed by ML using the software PHYML that generated 200 trees. The consensus phylogenetic tree was then obtained (CONSENSE). For NJ, data were analyzed with PROTDIST that generated 1000 distance matrices. The program NEIGHBOR was then employed to generate 1000 trees. Finally, the consensus phylogenetic tree was obtained using CONSENSE. Trees were drawn using the TreeViewX program *v*0.5.0. Presence of known domains in the predicted protein sequence of Senegalese sole eEF1As was determined by scanning in the PROSITE database [92].

### RNA isolation and gene expression analysis

Homogenization of juvenile tissues and larvae was carried out using the Lysing Matrix D (Q-BioGene) for 40 s at speed setting 6 in the Fastprep FG120 instrument (Bio101). Total RNA was isolated from 50 mg of pooled larvae using the RNeasy Mini Kit (Qiagen). All RNA isolation procedures were performed in accordance with the manufacturer's protocol. In all cases, total RNA was treated twice with DNase I using the RNase-Free DNase kit (Qiagen) for 30 min in order to avoid amplification of genomic DNA. RNA sample quality was checked using Experion (Bio-Rad), and quantification was performed spectrophotometrically. Total RNA (1 μg) from each sample was reverse-transcribed using the iScript™ cDNA Synthesis kit (Bio-Rad). Lack of genomic DNA contamination was confirmed by PCR amplification of RNA samples in the absence of cDNA synthesis.

Real-time analysis was carried out on an iCycler (Bio-Rad). Matching oligonucleotide primers were designed using the Oligo *v*6.89 software (Medprobe). To confer specificity, each Senegalese sole *eEF1A *primer pair (Table 4) was designed using as template the corresponding 3'-UTR, where the high sequence variability made impossible to correctly align them. In addition, amplification of specific *SseEF1A *encoding cDNA fragments was verified by direct sequencing of PCR products in a previous assay. Real-time reactions were accomplished in a 25 μl volume containing cDNA generated from 10 ng of original RNA template, 300 nM each of specific forward (F) and reverse (R) primers, and 12.5 μl of iQ™ SYBR Green Supermix (Bio-Rad). The amplification protocol used was as follows: initial 7 min denaturation and enzyme activation at 95°C, 40 cycles of 95°C for 15 s, and 70°C for 30 s. Each assay was done in duplicate. For normalization of cDNA loading, all samples were run in parallel using the housekeeping genes *GAPDH2 *[63] or *Ubiquitin *as loading standards for larval development and tissues, respectively (Table 4). To estimate efficiencies, a standard curve was generated for each primer pair based on known quantities of cDNA (10-fold serial dilutions corresponding to cDNA transcribed from 100 to 0.01 ng of total RNA). All calibration curves exhibited correlation coefficients higher than 0.99, and the corresponding real-time PCR efficiencies were in the range 0.92–0.98. Relative mRNA expression was determined using the 2^-(ΔΔCt) ^method [93]. To compare the amount of each of the Senegalese sole transcripts, the relative mRNA expression was determined by subtracting the Ct value of *GAPDH2 *or *Ubiquitin *mRNA from the Ct value of the target mRNA. Data were then expressed as the ratio (calculated using 2^-(ΔCt)^) of target mRNA to reference (*GAPDH2 *or *Ubiquitin*) mRNA. Owing to the small differences among amplicons both in size (62–142) and %GC (52.8, 58.1, 52.4, 46.7 and 67.8% for *SseEF1A1*, *SseEF1A2*, *SseEF1A3*, *SseEF1A4 *and *Sse42Sp50*, respectively), the relative sensitivity factor K_RS _was assumed to be one [94].

**Table 4:** Primers used for real-time gene expression analysis. The amplicon size generated by each primer pair is shown.

Target	Primers		Fragment size (bp)
		
	Primer pair name	Sequence	
*SseEF1A1*	SseEF1•1	5'-GATTGACCGTCGTTCTGGCAAGAAGC-3' (F)	142
	SseEF1•2	5'-GGCAAAGCGACCAAGGGGAGCAT-3' (R)	
*SeeEF1A2*	SseEF2•1	5'-TGGAAATGTGGCCGGTGACAGCA-3' (F)	124
	SseEF2•2	5'-GCAATCCAGAACGGGGGCGTA-3' (R)	
*SseEF1A3*	SseEF3•1	5'-GACCGTCGTAGTGGTAAAGCCCTGGAA-3' (F)	122
	SseEF3•2	5'-GGGGGAAACTCTGCAAACGCCTCA-3' (R)	
*SseEF1A4*	SseEF4•1	5'-GCCGTCGGTGTCATCAAGAAAGTCG-3' (F)	135
	SseEF4•2	5'-GAGATAAATCCTAATGTGGCTCCGTTGTCG-3' (R)	
*Sse42Sp50*	Sse42Sp50•1	5'-CCGGCTACTCCCCTGTGCTGGACT-3' (F)	62
	Sse42Sp50•2	5'-CAGCTCGGCAAAGCGGCAGGT-3' (R)	

Results were expressed as mean ± SEM. Comparisons among groups were carried out with one-way analysis of variance (ANOVA), followed by a Tukey test for identification of the statistically distinct groups. Significance was accepted for *P *< 0.05.

## Abbreviations

dat, days after TU treatment

datt, days after T4 treatment

DPH, days post hatch

ML, maximum likelihood

NJ, neighbor-joining

SEM, standard error of the mean

THs, thyroid hormones

TU, thiourea

## Authors' contributions

EA performed the Senegalese sole cultures and hormone exposures, as well as larval and tissue samplings. CI and MM performed the relative quantitation assays and wrote the manuscript. JP-C and MM designed the experiments, and participated in manuscript preparation. All authors read and approved the final manuscript.
